# Compliance with the 2021 surviving sepsis campaign Hour-1 Bundle and impact on patient outcomes in a resource-limited setting: A quality improvement initiative in a referral hospital in Rwanda

**DOI:** 10.1371/journal.pgph.0005091

**Published:** 2026-11-05

**Authors:** Jean de Dieu Mahoro, Jean Paul Mvukiyehe, Elisabeth Riviello, Theogene Twagirumugabe

**Affiliations:** 1 University Teaching Hospital of Butare, Huye, Rwanda; 2 College of Medicine and Health Sciences, University of Rwanda, Kigali, Rwanda; 3 Division of Pulmonary, Critical Care, and Sleep Medicine, Beth Israel Deaconess Medical Center and Harvard Medical School, Boston, Massachusetts, United States of America; National Center for Chronic and Noncommunicable Disease Control and Prevention, Chinese Center for Disease Control and Prevention, CHINA

## Abstract

Sepsis and septic shock are common life-threatening conditions associated with high mortality globally. Compliance with the Society of Critical Care Medicine’s Hour-1 Bundle for sepsis management has the potential to improve outcome, but the bundle is infrequently applied in resource-constrained settings. We assessed level of compliance with the Hour-1 bundle among patients diagnosed with sepsis and septic shock at the Accident and Emergency Department (AED) or Intensive Care Unit (ICU) of University Teaching Hospital of Butare, Rwanda. We conducted training for nurses and doctors in the AED and ICU, with the goal of improving compliance. We re-assessed bundle compliance one month after the training. We used univariate and multivariate analyses to evaluate the association of compliance and fluid resuscitation with hospital mortality while adjusting for severity of illness and other covariates. Our finding showed that seventy-eight patients were enrolled during the pre-intervention period, and 82 in post-intervention. The overall compliance with at least 4 elements of the Hour-1 bundle before the intervention was 14%, and 76% after the intervention (p < 0.001). Mortality was lower in the post-intervention period than in the pre-intervention period (51.2% vs 74.4%; p = 0.003). Non-survivors had a significantly lower bundle compliance than survivors (35% vs 80%; adjusted OR 0.18, 95% CI 0.05-0.57; p = 0.004). Administered 2 liters of IV fluids and beyond during the first hour was independently associated with higher mortality (adjusted OR 19.38, 95% CI 5.63-66.75; p **<** 0.001). Findings from this Quality Improvement project suggest that compliance with the Hour-1 sepsis bundle in a resource-constrained setting can be improved with training, can improve outcome.. Administration of ≥2 liters during the first hour in those was independently associated with a higher in-hospital mortality, although bundle-compliant resuscitation was not. More research is needed, both on the implementation of sepsis bundles in resource-constrained settings, to assess if all bundle elements are as effective in these settings as recommended in the international guidelines.

## Introduction

Sepsis is a dysregulated host response to infection [1], and has been designated by the World Health Organization (WHO) as a global health priority due to its very high mortality rate [2]. An estimated 85% of incident sepsis cases worldwide occur in low, lower-middle, and middle-income countries [3]. In Rwanda, a low-income country (LIC), sepsis and septic shock are major causes of morbidity and mortality. In a study conducted in the intensive care units (ICUs) of two public referral hospitals in Rwanda in 2016, sepsis was the leading cause of admission to the ICU; of patients enrolled in that study, those suspected or diagnosed with infection within 24 hours of ICU admission had a 2.14 increased odds ratio of death compared to patients without infection (4). Another study conducted in these same public referral hospitals in Rwanda in 2021 demonstrated that mortality associated with sepsis and septic shock was 51.4%, and 82.9% respectively [4]. This is much higher than the 38% mortality found in a meta-analysis of studies from North America and Europe among patients diagnosed with septic shock in ICUs [5]. This discrepancy in mortality may be due in part to differences in quality of care for septic patients, including early resuscitation, timely administration of appropriate antibiotics, and use of vasopressors [6–9]. However, prior trials have demonstrated different responses in high- and low-resource settings to liberal versus conservative fluid resuscitation; outcomes with liberal fluid resuscitation are similar to those with conservative resuscitation in high-income countries (HICs), but outcomes are worse with liberal fluid resuscitation in LICs [6,10,11].

Evidence for sepsis bundles that was generated in high-resource settings may not translate directly to low-resource settings, because bundle elements were largely developed and validated where point-of-care lactate testing, continuous hemodynamic monitoring, and intensive care capacity for managing fluid overload are routinely available. The same interventions, including fluid resuscitation, may have different physiologic effects and risk-benefit profiles in patients with a different burden of comorbid anemia, malnutrition, and cardiopulmonary disease, and in settings where the capacity to detect and treat complications, such as fluid overload, is limited [11–13]. Moreover, time to access care is often delayed in resource-limited settings; the same interventions later in a disease course may also have different outcomes.

The Society of Critical Care Medicine (SCCM) has published a sepsis Hour-1 Bundle that consists of five components: lactate measurement, blood cultures prior to antibiotic administration, administration of broad-spectrum antibiotics, initiating rapid administration of 30 ml/kg of crystalloid fluid if there is hypotension or lactate>4 mmol/L, and vasopressor initiation if there is hypotension [14–17]. In a study in the Netherlands, and another study in Japan, compliance with the bundle was associated with improved hospital survival [18,19]. The bundle is a standard practice for sepsis management in many highly-resourced areas of the world, but it is uncommonly used in resource-constrained settings. Little is known about the feasibility of implementing the bundle, or about its potential impact on patient outcomes in resource-constrained settings. Nor is it understood whether bundle-compliant fluid resuscitation is associated with better or worse outcomes. We measured compliance with the bundle at baseline in a Rwandan referral hospital Accident and Emergency Department (AED) and ICU; implemented bundle training for nurses and doctors in the AED and ICU; and then re-assessed compliance four weeks after the training. We also sought to understand the relationship between overall bundle compliance, bundle component compliance, and fluid resuscitation amount, with hospital mortality.

## Materials and methods

### Ethics statements

The study was approved by the Institutional Review Board of the University Teaching Hospital of Butare (CHUB) (Ref-REC/UTHB/048/2021) and adhered to the Declaration of Helsinki. Patients or next of kin provided a written consent prior to their enrollment. The privacy and confidentiality of data were ensured; data were collected from medical records and charts as well as observation at bedside. Hard copies of collected data were stored in a closed cupboard accessible only to the PI and research assistants, and they were entered into Excel by the PI. The dataset was password-protected on the PI’s computer, with a backup on a secure google drive.

### Design and study participants

We performed a prospective cohort study to assess compliance with the SCCM Hour-1 bundle before and after training of healthcare providers, and to assess for an association between bundle compliance and hospital mortality. The study is reported in accordance with the STROBE (Strengthening the Reporting of Observational Studies in Epidemiology) guidelines for observational research [20]. The study was conducted in the AED and ICU of the University Teaching Hospital of Butare (CHUB), Rwanda. Patients diagnosed with sepsis by treating clinicians in the AED or ICU were screened for enrollment in the study. During daytime hours in the pre- and post-intervention periods, the Principal Investigator and two research assistants screened patient charts and observed patient care, to locate instances of new diagnoses of sepsis as determined by clinicians in the AED and ICU. Once identified, the study staff further screened the patients for eligibility, by assessing whether they met criteria for sepsis using SCCM recommendations: suspected or confirmed infection; and with evidence of organ dysfunction as evidenced by Modified Early Warning Score (MEWS) ≥ 5, Sequential Organ Failure Assessment (SOFA) score ≥2 or a change of initial SOFA by more than 2 points [21,22] In practice, the team used a checklist with each of these scores, and confirmed eligibility when any of the score cutoffs were met; SOFA was never used for initial inclusion, as laboratory values were not back at the time of screening. Eligible patients or their next of kin were approached to provide a written informed consent.

The study was composed of three phases. Phase I included screening, enrolment, and data collection for septic patients before the intervention. Phase II consisted of a two-month-long period of training of all healthcare providers in the AED and ICU on sepsis management using a local guideline contextualized from the Surviving Sepsis Campaign Guidelines [22], including the Hour-1 sepsis bundle. Phase III consisted of screening, enrolment, and data collection for septic patients after the intervention. Phase III was initiated four weeks after the last training session.

### Intervention

A local guideline for management of sepsis and septic shock was developed based on the Hour-1 Bundle from SCCM [14,22] All healthcare providers from AED and ICU were trained on it with the following learning objectives:

- Timely recognition of sepsis using the SIRS, SOFA, qSOFA, MEWS, and NEWS scores where applicable, using any of these tools as clinical data is available. The training included handouts with the score elements and cutoffs;
- Adequate fluid resuscitation and monitoring within the first hour of recognition of sepsis;
- Timely identification of patients in shock, and use of vasopressors when fluid resuscitation does not allow achievement of an adequate tissue perfusion (Mean Arterial Pressure (MAP) ≥65 mmHg);
- Early recognition and monitoring of the adequacy of tissue perfusion in patients with sepsis or septic shock by measuring and interpreting serum lactate level;
- Early administration of appropriate antimicrobials, with the goal to obtain relevant cultures prior to antibiotic administration, and based on the study site (CHUB) empiric antibiotic guidelines.

Two one-hour training sessions included all anesthesiologists, anesthesia residents, general practitioners, and nurses working in the ICU. An additional two one-hour sessions included all nurses and general practitioners in the AED. The trainings were all led by the Principal Investigator (JDDM), and the training materials were given to clinicians. A summary algorithm was displayed in the department’s physician work stations, nurse work stations, and triage rooms as an ongoing reminder after the training.

### Sample size

We hypothesized that the mortality rate was as high as 82% based on a previous study [4], and we projected an absolute reduction of this mortality by 20% after the training intervention on the SCCM Hour-1 bundle. With a study power of 80% and type-1 error of 0.05, the calculated minimum sample required per group was 78 participants.

### Data collection

The data collected included patients’ baseline characteristics, compliance with blood sampling and infection source site sampling for culture prior to antibiotic administration, amount of intravenous crystalloids given within one hour of sepsis recognition, administration of appropriate empiric antimicrobial drug administration within one hour of sepsis recognition, administration of vasopressors within the first hour when administered fluids were ineffective to achieve a MAP ≥ 65 mmHg, and laboratory request for lactate level during the first hour of sepsis recognition. Data collection was done at bedside, and included observation both of chart documentation, as well as observed activities performed by the physicians and nurses.

MEWS and SOFA scores for each patient were collected; many times data was not fully available in the first hour after sepsis recognition, especially laboratory values. Research assistants returned to the patient’s chart after the initial one-hour period to complete the scores as the data became available. Research assistants recorded whether SOFA was calculated from complete data, or whether a variable within the SOFA score was missing. The team also recorded discharge date and vital status at discharge. The Research assistants and PI met weekly to review enrollment numbers and assess data quality.

### Definitions and data analysis

We used a cutoff of >5 for both the MEWS and SOFA scores [23,24]; missing data for either of these scores was imputed as normal but we verified the association for data with no imputed SOFA scores. We reported the proportion of participants requiring imputation for their SOFA scores by study period. When there was no arterial blood gas (ABG) available at the site, we imputed PaO2/FiO2 from Spo2/ FiO2 [25]. We defined “complete” bundle compliance as compliance with at least four of the five elements of the bundle. We chose a threshold of ≥4 of 5 bundle elements to define bundle compliance because lactate measurement is a bundle element that had only intermittent availability with point-of-care testing at our site. Requiring strict 5/5 compliance would have systematically misclassified many otherwise well-managed cases as non-compliant due to a resource limitation unrelated to individual adherence to the protocol.

Descriptive statistics were used to determine the frequency distribution of all quantitative variables including completion of Hour-1 bundle components and mortality. Comparison between categorical variables was done by using Chi-square or Fisher exact tests. The hospital mortality rate was the primary outcome; we compared mortality between Phase 1 and 3, and also between those with complete bundle compliance and those without. We performed binary univariate analyses to look for associations between baseline characteristics and bundle compliance with mortality. Variables with p < 0.25 were selected for multivariable modeling, and location of sepsis diagnosis was included in the multivariable modeling as well.

A multivariable logistic regression model was constructed including location of sepsis diagnosis, MEWS score, type of the clinical condition (medical versus surgical), source of infection, and volume of fluid administered. Adjusted odds ratios (ORs) with 95% confidence intervals (CIs) were reported. A p-value < 0.05 was considered statistically significant.

We performed two sensitivity analyses. First, we calculated propensity score by using location, types of condition (medical vs surgical), source of infection, MEWS and compliance to one hour bundle. We computed an inverse probability weighting (IPW) for each participant to create a balanced pseudo-population. A doubly robust regression model was then fitted, adjusting for the same covariates as the primary analysis while weighting observations by IPW. Results were compared with the primary multivariable analysis to assess consistency of findings. Statistical significance was defined as p < 0.05. Second, we repeated the primary univariate and multivariate analyses restricted to the subset of patients with complete data for all model covariates (i.e., no imputed SOFA components; N = 68) (Table E in S1 Appendix and Table F in S1 Appendix). All analyses were done with SPSS version 25 IBM, Chicago-IL, USA

## Results

### Enrollment

Phase 1 occurred from December 1, 2021 to August 19, 2022; phase 2 occurred from September 1, 2022 to November 1, 2022; and phase 3 occurred from November 28, 2022 to August 25, 2023. During Phase 1, 81 patients were eligible, and of them, 78 patients consented and were enrolled; three patients died within less than one hour of admission (Fig 1). During Phase 3, 84 patients were eligible, and 82 patients were enrolled; two patients died within the first hour of admission. Of those enrolled, 43 patients were in the AED and 35 in the ICU in phase 1; 38 patients were in the AED and 44 patients in the ICU in phase 3. All eligible patients who survived their first hour after sepsis recognition provided their consent to participate in the study (either directly or via next of kin). All patients designated as septic by clinicians were confirmed to be septic by the research assistants using SCCM criteria (Fig 1).

**Fig 1:**
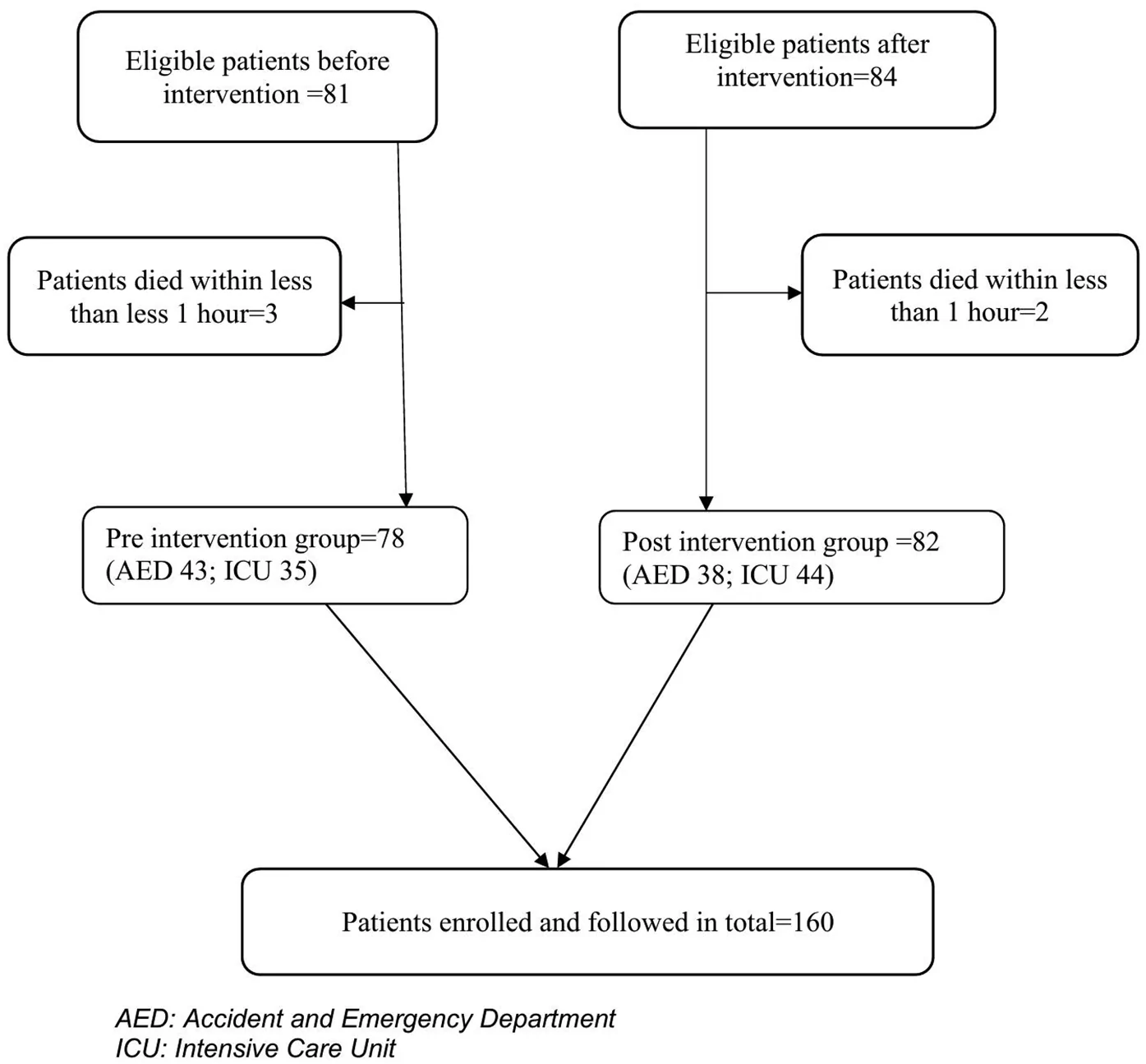
Enrolment.

### Pre and post-intervention period baseline patient characteristics

In total, 160 participants were recruited for the study. Of these, 75 (46.9%) were female, and the median age with interquartile range was 49 (3363.7) years (Table 1). More than half of participants (84 participants, 52.5%) were admitted due to surgical conditions, and almost all of those (82, 51.2% of the total), had intrabdominal sepsis. The patients in Phase 1 were more severely ill than those in Phase 2: the median (IQR) SOFA score before the intervention was 11 (8–13) compared with 5 (3.7-10.7) during the post-intervention period (p < 0.001). Similarly, the median (IQR) MEWS before the intervention was 7 (5–9) whereas it was 3 (2-7.2) in the post-intervention period (p < 0.001). Whether the SOFA was imputed versus complete was not different between the two periods. (p = 0.36) (Table A in S1 Appendix) When SOFA required imputation, it was always due to missingness in the bilirubin variable. The input variables for MEWS had complete data for every participant

**Table 1:** Baseline demographic and clinical characteristics before and after intervention.

*Variable*		*All periods*	*Before intervention* *N = 78*	*After intervention N = 82*	*p value*
Sex	Female N (%)	75 (46.9)	34 (43.6)	41 (50.0)	0.417
Age	Median (IQR)	49 (33-63.7)	52 (35.7-65.5)	44 (30.0-63.2)	0.108
MEWS	Median (IQR)	5 (3-9)	7 (5-9)	3 (2-7.25)	<0.001
SOFA	Median (IQR)	9 (4-13)	11 (8-13)	5(3.7-10.7)	<0.001
Surgical vs medical condition	Patient with surgical condition N (%)	84 (52.5)	54 (69.2)	30 (36.6)	<0.001
Source of infection	CNS; N (%)	25 (15.6)	7 (9.0)	18 (22.0)	<0.001
	Respiratory; N (%)	44 (27.5)	11 (14.1)	33 (40.2)	
	Abdomen; N (%)	82 (51.2)	53 (67.9)	29 (35.4)	
	Other; N (%)	9 (5.6)	7 (9.0)	2 (2.4)	
Location	ICU N (%)	79 (49.4)	35 (44.9)	44 (53.7)	0.267
	A&E N (%)	81 (50.6)	43 (55.1)	38 (46.3)	

*MEWS: Modified Early Warning Score; SOFA: Sequential Organ Failure Assessment; CNS: Central Nervous System. Other includes bacteremia of unknown source and urinary tract infection.*

### Compliance with Hour-1 Bundle in the two periods

Overall, compliance with at least 4 elements of the bundle occurred in 14.1% of cases during the pre-intervention period and 75.6% in post-intervention period (p < 0.001) (Table 2, Fig A in S1 Appendix). The compliance in measuring lactate before and after the intervention was similar in the two periods (25.6% vs 29.3%, p = 0.609). However, the compliance with obtaining culture before antibiotics was significantly improved after the intervention (33.3% vs 90.2%, p < 0.001). The administration of appropriate broad-spectrum antibiotics was also much higher in the post-intervention period (29.5% vs 81.7%, p < 0.001). The compliance with fluid resuscitation by the guideline was numerically higher in Phase 3 than Phase 1, but it did not reach statistical significance (78.2% vs 89%; p = 0.065). The administration of vasopressors if indicated by the clinical conditions was adequate in 52.6% cases vs 86.6% (p < 0.001) during the pre-intervention and post-intervention periods respectively. In addition, the time to starting a vasopressor and time to administering an antibiotic were statistically different, with pre-intervention and post-intervention respective median (IQR) and p-values: 65 minutes (55–71) versus 55 minutes (45–65), p < 0.001; and 84 minutes (50–95) versus 40 minutes (30–50); p = 0.005 Fig A in S1 Appendix).

**Table 2:** Compliance with Hour-1 bundle in the two periods.

Component of the bundle	Before intervention N(%)	After intervention N(%)	P-value
Measure lactate level	20 (25.6)	24 (29.3)	0.723
Obtain sample for culture before empiric antibiotics	26 (33.3)	74 (90.2)	<0.001
Administer broad-spectrum antibiotics	23 (29.5)	67 (81.7)	<0.001
Fluid administration if indicated	61 (78.2)	73 (89.0)	0.086
Applying vasopressor if indicated	41 (52.6)	71 (86.6)	<0.001
Overall compliance (At least 4 bundle elements completed)	11 (14.1)	62 (75.6)	<0.001

*Both fluid administration and vasopressor administration were counted as compliant if: 1) they were indicated and given, or 2) they were not indicated and not given.*

### Patient outcomes in the two periods

The overall hospital mortality among 160 study participants was 62.5% (Table C in S1 Appendix). Mortality was higher in the pre-intervention period than in the post-intervention period (74.4% vs 51.2%, p = 0.003).The median hospital length of stay among survivors was not significantly different between the two periods. It was 6.5 (IQR 3.2-13.2) days and 9.5 (IQR 7.2-12.7) days in the pre and post-intervention periods, respectively p = 0.111(Table C in S1 Appendix).

### Factors associated with mortality

In all patients from Phase 1 and 3, we evaluated the association of sex, age, MEWS, SOFA score, surgical vs medical disease, administered fluids ≥ 2 liters in the first hour, source of infection, compliance with Hour-1 bundle, and compliance with each component of the Hour-1 bundle, with hospital mortality (Table 3). In univariate analyses, MEWS>5, SOFA>5, surgical disease, ≥ 2 liters administered, and non-respiratory source of infection, were all found to be associated with hospital mortality, with p < 0.25. In addition, overall bundle compliance and compliance with each element of the bundle were found to be associated with lower mortality, with p < 0.25. There appears to be a dose-response relationship between compliance with bundle elements and mortality: increasing from 1 to all 5 of the components of the bundle being performed was associated with a stepwise decrease in mortality (Fig 2).

**Table 3:** Univariate analysis associated with in-hospital mortality.

Variable, N (%)		Not survived N=100	Survived N=60	P value
Location	ICU	49(49)	30 (50)	0.903
	A&E	51(51)	30(50)	
Gender	Female	47 (47)	28 (46.7)	0.967
Age	Median (IQR)	49 (31.3-65.3)	48.5 (34.3-63.8)	0.418
MEWS	MEWS>5	68 (68)	8 (13.3)	<0.001
SOFA	SOFA >5	85 (85)	17 (28.3)	<0.001
Surgical or medical conditions	Patient with medical conditions	43 (43)	33 (55)	0.141
Volume of fluids administered	Fluid >=2 liters in first hour	68 (68)	7 (11.7)	<0.001
Source of infection	CNS and other	29 (29)	5 (8.3)	<0.001
	Respiratory	15 (15)	29 (48.3)	
	Abdomen	56 (56)	26 (43.3)	
Overall Bundle compliance	Compliance with at least 4 bundle elements	35 (35)	48 (80)	<0.001
Antibiotic administration	Compliance	43 (43)	47 (78.3)	<0.001
Microbiology sample collection	Compliance	53 (53)	47 (78.3)	0.001
Administration of 30 cc/kg	Compliance	79 (79)	55 (91.7)	0.035
Vasopressor if needed	Compliance	55 (55)	57 (95)	<0.001
Lactate measured	Compliance	23 (23)	21 (35)	0.100

*MEWS: Modified Early Warning Score; SOFA: Sequential Organ Failure Assessment; CNS: Central Nervous System. Other includes bacteremia of unknown source and urinary tract infection.*

*CNS and other are combined given the low number in each of these categories.*

**Fig 2:**
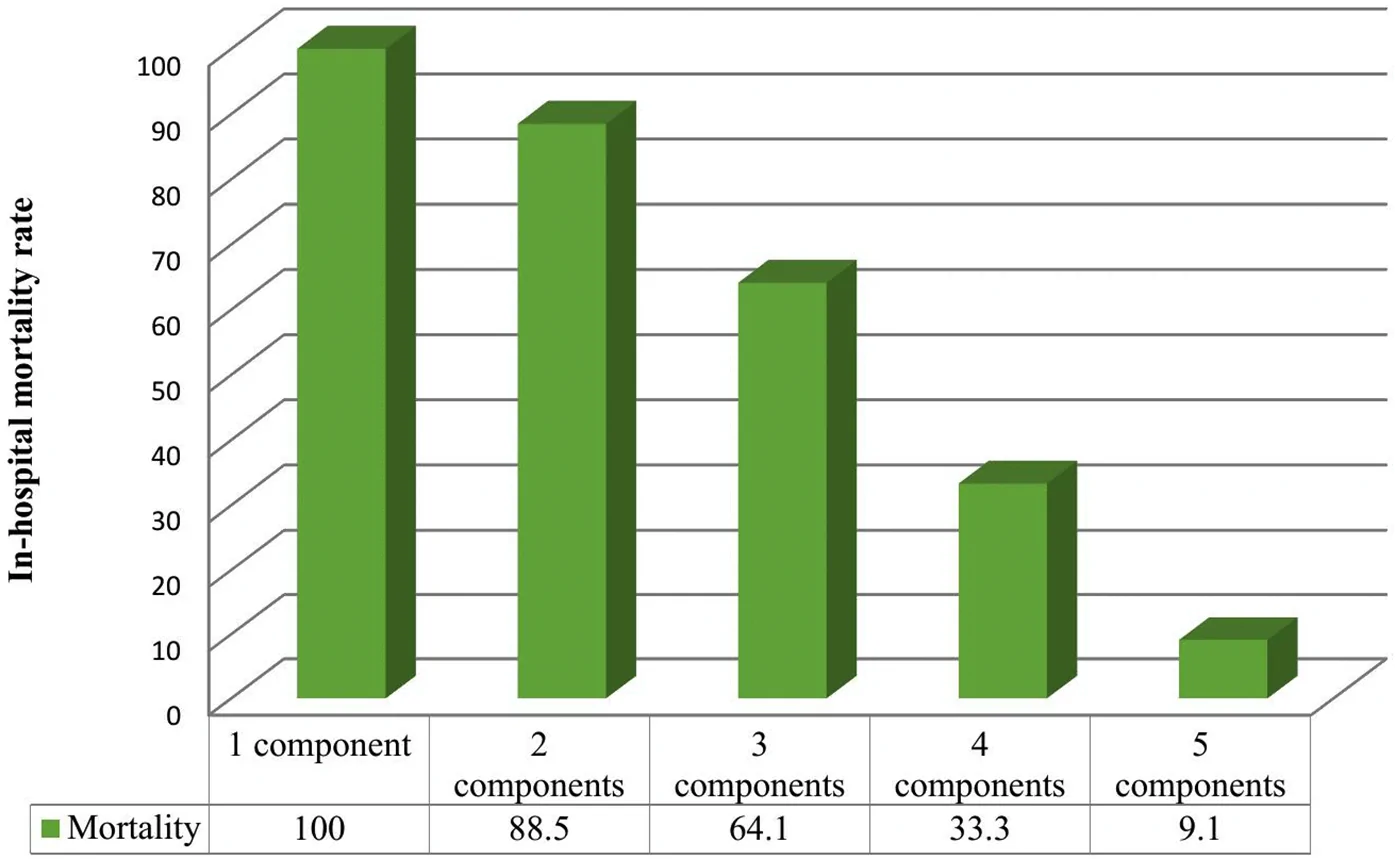
Bundle compliance rate and survival (n=160).

We used variables with association with mortality of p < 0.25 from the univariate analyses in a multivariate logistic regression analysis. We included overall compliance but not compliance with each component of the bundle, given the overlap of variables. We include treating location given its face-value likelihood of impacting outcome, though its P value was not significant in univariate analyses. We used MEWS>5 rather than SOFA>5 as the severity of illness score in the multivariate analysis because we had complete data for calculation of MEWS for all participants. MEWS>5 and administration of IV fluids ≥ 2 liters in the first hour were independently associated with hospital mortality with adjusted odds ratios (AOR): 7.55 (95% CI 2.40-23.73, p = 0.001) and 19.38 (95% CI 5.63–66.75, p < 0.001) respectively (Table 4). Overall compliance with 4 or more bundle components and having sepsis with a respiratory source were independently associated with reduced hospital mortality (AOR 0.18, 95% CI 0.05-0.57, p = 0.004; AOR 0.03, 95% CI 0.00-0.15, p < 0.001 respectively.)

**Table 4:** Multivariate analysis of factors associated with hospital mortality.

Variable, N (%)		Not survived N=100	Survived N=60	P value	AOR [95% CI]
MEWS	MEWS>5	68 (68)	8 (13.3)	0.001	7.55[2.40-23.73]
Location	ICU	49(49)	30 (50)	0.462	1.48[0.52-4.21]
Surgical or medical conditions	Patient with medical conditions	43 (43)	33 (55)	0.57	1.86[0.21-15.90]
Volume of fluids administered	Fluid >=2 liters in first hour	68 (68)	7 (11.6)	<0.001	19.38 [5.63-66.75]
Source of infection	CNS and other	29 (29)	5 (8.3)	Reference	
	Respiratory	15 (15)	29 (48.3)	<0.001	0.03[0.00-0.15]
	Abdomen	56 (56)	26 (43.3)	0.194	0.21[0.02-2.19]
Overall Bundle compliance	Compliance to at least 4 bundle elements	35 (35)	48 (80)	0.004	0.18[0.05-0.58]

*MEWS: Modified Early Warning Score; CNS: Central Nervous System. Other includes bacteremia of unknown source and urinary tract infection.*

*CNS and other are combined given the low number in each of these categories.*

*AOR: adjusted odds ratio*

We computed the IPW for the doubly robust regression model, and some differences emerged. Whereas the multivariate analysis suggested ICU location was not significantly associated with mortality, doubly robust regression identified ICU care as a significant risk factor for in-hospital mortality (AOR = 4.58, p = 0.007). Similarly, effect sizes for fluid administration (AOR = 77.33 vs 19.38) and bundle compliance (AOR = 0.13 vs 0.18) were more pronounced in the doubly robust analysis, suggesting the primary analysis may have underestimated these relationships due to confounding. However, the very wide confidence interval around the fluid-administration estimate (95% CI 16.49–362.62) reflects substantial statistical imprecision and possible model instability; the magnitude of this estimate should not be interpreted as precise, and the finding should be understood as an association, for which residual confounding by indication remains possible (Table 5).

**Table 5:** Sensitivity analysis of factors associated with in-hospital mortality by using doubly robust regression.

Variable	Category	Not survived N=100	Survived N=60	P value	AOR [95% CI]
MEWS Score	MEWS>5	68 (68)	8 (13.3)	0.001	13.2 [4.12-42.42]
Location	ICU	49(49)	30 (50)	0.007	4.58 [1.50-13.96]
Surgical or medical conditions	Surgical	43 (43)	33 (55)	0.467	2.40 [0.22-25.64]
Volume of fluid administered	Fluid ≥2 Lin 1st hour	68 (68)	7 (11.6)	<0.001	77.33 [16.49-362.62]
Source of Infection	CNS & Others	29 (29)	5 (8.3)	Ref	
	Respiratory	15 (15)	29 (48.3)	<0.001	0.00 [0.00-0.03]
	Abdomen	56 (56)	26 (43.3)	0.161	0.66 [0.01-2.04]
Overall Compliance	Compliance to at least 4 bundle elements	35 (35)	48 (80)	0.017	0.13 [0.02-0.69]

*MEWS: Modified Early Warning Score; CNS: Central Nervous System. Other includes bacteremia of unknown source and urinary tract infection.*

*CNS and other are combined given the low number in each of these categories.*

*AOR: adjusted odds ratio*

The Hosmer and Lemeshow Test of the model confirms that there is no significant difference between the observed and predicted values, as the chi-square value of 4.87 with 8 degrees of freedom has a p-value of 0.675, which is greater than 0.05(Table D in S1 Appendix). This suggests that the model is a good fit for the data.

In a sensitivity analysis restricted to the N = 68 patients with complete data for all model covariates (i.e., no imputed SOFA components), findings were broadly consistent with the primary analysis. Administration of ≥2 liters of fluid in the first hour remained strongly associated with higher in-hospital mortality (AOR 14.23, 95% CI 2.13–95.12, p = 0.006), and overall compliance with at least 4 bundle elements remained associated with a lower in-hospital mortality (AOR 0.08, 95% CI 0.01–0.54, p = 0.010). ICU location (AOR 3.01, 95% CI 0.50–18.26, p = 0.230) and medical (versus surgical) condition (AOR 1.29, 95% CI 0.30–5.58, p = 0.730) remained non-significant, as in the primary analysis (Tables E and F in S1 Appendix).

## Discussion

There are almost 49 million cases of sepsis each year, and 11 million sepsis-related deaths; this represents over 19.7% of all global deaths annually [3]. Incidence of sepsis and sepsis-related mortality remain enormously high in LICs, and in sub-Saharan African countries in particular [26]. SCCM’s international guidelines, recently updated under the Surviving Sepsis Campaign, have demonstrated effectiveness in reducing mortality in resource-rich settings [18,19].

Our study demonstrates that training on the Hour-1 sepsis bundle is feasible, and was associated with a marked increase in compliance with at least four of five bundle elements. Our adjusted analyses also suggest that increased compliance with the bundle is associated with decreased mortality. While the large reduction in mortality observed between the pre- and post-intervention periods is likely impacted by lower severity of illness in the second period and secular trends, we found a smaller but still significant lower mortality when controlling for severity of illness; we also found an association between bundle compliance and lower mortality when assessing all participants in both periods as one cohort.

Of note, the component variable of measuring lactate did not statistically improve between periods; this is likely due to inconsistent availability of point of care arterial blood gas analysis with lactate [6].

In addition, bundle-consistent intravenous fluid resuscitation of 30 ml/kg was associated with lower mortality in univariate analysis (Table 3), while intravenous fluid resuscitation of 2 liters or more was associated with higher mortality in both univariate and multivariate analyses (Tables 3 and 4). Given the lower weight of many of our patients, 30 ml/kg is often much less than 2 liters. We examined both compliance with 30 ml/kg resuscitation and incidence of ≥ 2 liters resuscitation because previous trials have demonstrated that liberal fluid resuscitation in both adults and children with sepsis in resource-constrained settings is associated with higher mortality [6,11]. Higher mortality with ≥ 2 liters fluids resuscitation is suggested by our findings, even when controlling for severity of illness, but residual confounding by indication cannot be entirely excluded, especially given that sicker patients are more likely to get more fluids. Our study also demonstrates that bundle-compliant 30 ml/kg resuscitation may be associated with lower mortality in LIC settings. Our findings highlight the potential benefit of weight-indexed and physiology-guided resuscitation in LIC settings, and underscore the need for international guidelines that incorporate evidence from trials from a variety of contexts and populations, including resource-constrained settings [27]. In addition, future studies with richer process evaluation and post-resuscitation data could strengthen causal inferences and guide context-specific care.

To our knowledge, this is the first study to evaluate the impact of compliance with the Hour-1 bundle on the outcome of patients admitted with sepsis in a resource-constrained setting. This is a pragmatic study reflecting real-world circumstances; our results, both in terms of feasibility of improving bundle uptake, and in the suggestion of improved outcomes with improved bundle compliance, are important for other clinicians in similar resource-constrained settings.

Our study has several limitations. First, hospital mortality is our primary outcome, and we do not have data on other important outcomes for patients with critical illness, including longer-term mortality and functional status. Second, there may have been some Hawthorne effect, particularly after the intervention when clinicians knew what elements were being observed; this may have inflated post-intervention compliance estimates. To limit this bias, research assistants were trained to observe unobtrusively and did not provide real-time feedback, coaching, or correction during observed encounters, and the same observation protocol was applied in both study periods. Of note, using bedside observation gave us very accurate data on bundle compliance in the first hour, which would be impossible to obtain without bedside presence. Third, we were only able to enroll patients during daytime hours; it is possible that we would see different results for patients enrolled during night. Nighttime presentations may differ from daytime presentations in case-mix, staffing ratios, and care processes, and this is an important selection bias that may limits the generalizability of our findings to nighttime sepsis care. Continuous enrolment should be a priority in future studies to ensure findings are representative of the full spectrum of sepsis presentations across all hours of the day. Fourth, we have used a before-and-after design, which is at risk for unmeasured confounding factors and the influence of secular trends. However, we used a multivariate logistic regression to control for factors most clearly associated with mortality, including illness severity scores, and we combined participants from both study periods so that the association between bundle compliance and mortality could be evaluated across a wide range of illness severity. Using two study periods in this way mitigates confounding related to study period to some extent, but it does not remove all confounding due to case mix, secular trends, or other temporal factors that may differ between the periods but the time difference between the two periods was of one month only, and no change in terms of infrastrucure and resources was operated to cast a substantial disparity. Regression to the mean and residual confounding are additional potential sources of error. Future research should employ cluster randomized controlled trials and interrupted time-series studies to further mitigate the influences of unmeasured confounders and secular trends. Fifth, some of our adjusted odds ratios, particularly for fluid administration, were very large with correspondingly wide confidence intervals, which may reflect instability due to the limited number of events in some covariate strata. To assess the robustness of this finding, we conducted two additional sensitivity analyses: one using inverse probability weighting with a doubly robust regression model, and another restricting the sample to patients with complete data for all model covariates (i.e., no imputed SOFA components; N = 68). Both yielded results consistent with the primary analysis, supporting the direction of the observed association. Finally, we assessed the post-intervention compliance with the bundle at four weeks from the intervention; we were unable to assess compliance later to understand longer-term retention of learning.

## Conclusion

Compliance with the Hour-1 bundle of the 2021 Surviving Sepsis Campaign is low in resource-limited settings. However, it was substantially improved by a brief training intervention of staff. The bundle compliance was inversely asssociated with in-hospital mortality. Notably, the impact of fluid resuscitation was sensitive to the particular amount administered, with bundle-compliant resuscitation associated with improved outcomes, but more liberal fluid resuscitation over 2 liters was associated with worse outcome. This insinuates that guidelines and bundles validated in a variety of contexts are needed. Our study demonstrates that it is possible to increase compliance with a bundle with relatively few resources, and increased compliance was associated with improved patient outcomes in this setting; however, given the non-randomized before-after design, this association should also not be interpreted as evidence of a causal effect.

## Supporting information

S1 Appendix Supporting figure and tables. Fig A. Level of compliance with each component of the Hour-1 bundle and overall, during the two periods. Table A. SOFA imputation and completeness by study period. Table B. Timing to antibiotic and vasopressor administration by study period. Table C. Patient outcomes by period Table D. Hosmer and Lemeshow test for IPW analysis. Table E. Complete-case sensitivity analysis: univariate analysis associated with in-hospital mortality (N = 68). Table F. Complete-case sensitivity analysis: multivariate analysis of factors associated with hospital mortality (N = 68).
(DOCX)

S1 Data Dataset in excel format. (XLSX)
