# Supplementary material for: Compliance with the 2021 surviving sepsis campaign Hour-1 Bundle and impact on patient outcomes in a resource-limited setting: A quality improvement initiative in a referral hospital in Rwanda
Source: PLOS Glob Public Health. 2026 Nov 5;6(11):e0005091. doi: 10.1371/journal.pgph.0005091 (PMC13739136; doi:10.1371/journal.pgph.0005091)
Supplement: Supplementary file 1 [file pgph.0005091.s001.docx]

**Appendix**

**S1 Fig. Level of compliance with each component of the Hour-1 bundle and overall, during the two periods**

|  | Before intervention | After intervention |
| --- | --- | --- |
| Measure lactate leve | 25.6% | 29.3% |
| Obtain sample for culture before antibiotherapy | 33.3% | 90.2% |
| Administer blood spectrum antibiotics | 29.5% | 81.7% |
| Fluid administration if indicated | 78.2% | 89% |
| Applying vasopressor if indicated | 52.6% | 86.6% |
| Overall compliance(at least 4 bundle elements completed) | 14.1% | 75.6% |

**S1 Table. SOFA imputation and completeness by study period**

| Variables | | Before intervention | After intervention | P value |
| --- | --- | --- | --- | --- |
| Imputed SOFA versus not imputed | Imputed | 42 (45.7) | 50 (54.3) | 0.362 |
|  | Not imputed | 36 (52.9) | 32 (47.1) |  |

Participants with imputed SOFA all had the variable bilirubin missing, and bilirubin was imputed to be normal for these participants. All other SOFA variables are available for all admitted patients.

**S2 Table. Timing to antibiotic and vasopressor administration by study period**

| Variables | | Before intervention | After intervention | P value |
| --- | --- | --- | --- | --- |
| Timing to antibiotic administration: In minutes | Median (IQR) | 84 (50-95) | 40 (30-50) | <0.001 |
| Timing to vasopressor administration: In minutes | Median (IQR) | 65 (55-71) | 55 (45-65) | 0.005 |

**S3 Table. Patient outcomes by period**

| **Variables** | **Overall** | **Before intervention** | **After intervention** | **P value** |
| --- | --- | --- | --- | --- |
| Mortality, N (%) | 100 (62.5) | 58 (74.4) | 42 (51.2) | 0.003 |
|  |  |  |  |  |
|  |  |  |  |  |
| Length of stay for survivors in days Median (IQR) | 9 (9-12.75) | 6.5 (3.2-13.2) | 9.5 (7.2-12.7) | 0.111 |

**S4 Table. Hosmer and Lemeshow test for IPW analysis**

| **Chi-square** | **df** | **P value** |
| --- | --- | --- |
| 7.86 | 8 | 0.447 |

**S5 Table. Complete-case sensitivity analysis: univariate analysis associated with in-hospital mortality (N=68)**

| **Variable, N (%)** |  | **Not survived N=54** | **Survived N=14** | **P value** |
| --- | --- | --- | --- | --- |
| Location | ICU | 26 (48.1) | 8 (57.1) | 0.765 |
|  | AE | 28 (51.9) | 6 (42.9) |  |
| Gender | Female | 25 (46.3) | 6 (42.9) | 1.000 |
| Age | Median (IQR) | 46 (34.7-64.0) | 39 (30.7-67.0) | 0.539 |
| MEWS | MEWS>5 | 45 (83.3) | 0 (0.0) | <0.001 |
| SOFA | SOFA>5 | 53 (98.1) | 9 (64.3) | 0.001 |
| Surgical or medical conditions | Patient with medical conditions | 24 (44.4) | 6 (42.9) | 1.000 |
| Volume of fluids administered | Fluid ≥2 liters in first hour | 37 (68.5) | 2 (14.3) | <0.001 |
| Source of infection | CNS and other | 16 (29.6) | 0 (0.0) | 0.031 |
|  | Respiratory | 10 (18.5) | 5 (35.7) |  |
|  | Abdomen | 28 (51.9) | 9 (64.3) |  |
| Overall Bundle compliance | Compliance with at least 4 bundle elements | 18 (33.3) | 12 (85.7) | 0.001 |
| Antibiotic administration | Compliance | 18 (33.3) | 11 (78.6) | 0.005 |
| Microbiology sample collection | Compliance | 27 (50.0) | 12 (85.7) | 0.018 |
| Administration of 30 cc/kg | Compliance | 42 (77.8) | 14 (100.0) | 0.059 |
| Vasopressor if needed | Compliance | 28 (51.9) | 14 (100.0) | 0.001 |
| Lactate measured | Compliance | 13 (24.1) | 4 (28.6) | 0.737 |

**S6 Table. Complete-case sensitivity analysis: multivariate analysis of factors associated with hospital mortality (N=68)**

| **Variable, N (%)** | | **Non- survivors N=54** | **Survivors N=14** | **P value** | **AOR [95% CI]** |
| --- | --- | --- | --- | --- | --- |
| Volume of fluids administered | Fluid ≥2 liters in first hour | 37 (68.5) | 2 (14.3) | 0.006 | 14.23 [2.13-95.12] |
| Overall Bundle compliance | Compliance to at least 4 bundle elements | 18 (33.3) | 12 (85.7) | 0.010 | 0.08 [0.01-0.54] |
| Location | ICU | 26 (48.1) | 8 (57.1) | 0.230 | 3.01 [0.50-18.26] |
| Surgical or medical conditions | Patient with medical conditions | 24 (44.4) | 6 (42.9) | 0.730 | 1.29 [0.30-5.58] |

*AOR: adjusted odds ratio. Model included fluid volume (≥2 vs <2 liters in the first hour), overall bundle compliance (≥4 vs <4 elements), location (ICU vs AED), and condition type (medical vs surgical); MEWS and source of infection, included in the primary (imputed-data) multivariate model, could not be included here due to the smaller complete-case sample.*
